# Associations Between Binge-Eating Symptoms and Chronotype Among Bariatric Surgery Candidates: Clinical Implications for Preoperative Assessment—A Cross-Sectional Study

**DOI:** 10.3390/jpm16010037

**Published:** 2026-01-07

**Authors:** Giovanna Lira Rosa Ciutti, Andréia Gomes Bezerra, Marcos Mônico-Neto, Lia Rita Bittencourt, Sergio Tufik, Gabriel Natan Pires, José Carlos Fernandes Galduróz

**Affiliations:** 1 Departamento de Psicobiologia, Universidade Federal de São Paulo, São Paulo 04024-002, Brazil; 2 Programa de Pós-Graduação Interdisciplinar em Ciências da Saúde, Universidade Federal de São Paulo, Santos 11015-020, Brazil; 3 Instituto do Sono, São Paulo 04024-002, Brazil; 4 Hospital Israelita Albert Einstein, São Paulo 05653-120, Brazil; 5 Faculdade Israelita de Ciências da Saúde Albert Einstein, São Paulo 05653-120, Brazil

**Keywords:** sleep, chronotype, eating disorders, obesity, anxiety, depression

## Abstract

The prevalence of binge-eating behavior among individuals with obesity is reported to be higher than in the overall population. Previous studies have suggested that chronotype (more specifically, eveningness) is associated with binge-eating symptoms; however, this association remains unclear among individuals with obesity. **Background/Objectives**: To evaluate the association between chronotype and binge-eating symptoms in adults with severe obesity undergoing preoperative evaluation for bariatric surgery. **Methods**: This cross-sectional study evaluated 100 adults with severe obesity undergoing multidisciplinary preoperative assessment at a bariatric surgery clinic. Binge-eating symptoms were assessed using the Binge-Eating Scale. Chronotype was evaluated using the Morningness–Eveningness Questionnaire. Other sleep parameters were subjectively assessed using the Insomnia Severity Index, the Epworth Sleepiness Scale, the Pittsburgh Sleep Quality Index, the Functional Outcomes of Sleep Questionnaire, and the Berlin questionnaire. Psychological aspects were assessed using the Depression, Stress, and Anxiety Scale (DASS-21). **Results**: Clinically relevant binge-eating symptoms were identified in 50% of the patients. Regarding chronotype, 16 patients were evening-types, 45 were intermediate types, and 39 were morning-types. The proportion of the clinical sample with moderate or severe binge-eating symptoms was equivalent among the three chronotypes (*p* = 0.794), with patients with no binge-eating symptoms accounting for around 50% of each group. There was no association between chronotype and the binge-eating score (*p* = 0.702). **Conclusions**: Despite the high prevalence of binge-eating symptoms and an overall negative sleep profile (composed of excessively daytime sleepiness, poor sleep quality, and a high risk of sleep apnea), chronotype does not appear to influence binge-eating symptoms in this clinical sample of adults with severe obesity evaluated for bariatric surgery. These findings suggest a limited utility of chronotype assessment for identifying vulnerability to binge-eating symptoms in the preoperative setting.

## 1. Introduction

The prevalence of binge-eating behavior is estimated to be up to 3% in the general population [1], although higher frequencies have been reported in recent studies. Binge-eating behavior is not restricted to the population living with obesity, but its incidence is certainly higher in this population, which makes treatment difficult and leads to additional complications. According to Duchesne et al. [2], 7.5% to 30% of patients with obesity suffer from binge-eating behavior, estimates that have been replicated by multiple studies [3,4], although even higher prevalence estimates of 64.8% have been reported in a Brazilian sample [1]. Multiple mental health comorbidities are also frequently associated with binge-eating symptoms, including anxiety, depression, alcohol abuse, overall psychological distress, and lower self-esteem [4,5,6]. Therefore, in the clinical management of obesity, particularly in specialized care settings, it is important to assess the co-occurrence of binge-eating symptoms, as this may be associated with neuropsychological characteristics that may interfere with treatment adherence and long-term outcomes [7].

Given the complex interplay between binge-eating symptoms and various psychological and behavioral factors, recent research has begun to explore the potential role of chronobiological patterns—specifically chronotype—as a contributing factor in disordered eating behaviors. Chronotypes are phenotypes related to the temporal organization of someone’s activities, including the preferred waking and sleeping times [8]. Chronotypes are usually regarded as a psychological trait, as a personal circadian preference for sleep, and the performance of daytime activities, although recent evidence suggests that a significant part of it is biologically determined [8,9]. In humans, there are three well-defined chronotypes—morning-types, intermediate types, and evening-types. Misalignment between an individual’s chronotype—the behavioral expression of their biological clock—and the external light–dark cycle has been associated with poorer cognitive and behavioral outcomes, as well as an increased vulnerability to mental health conditions [10]. In particular, evening-type individuals often present less favorable behavioral and psychological profiles, largely due to chronic circadian misalignment between internal biological rhythms and social demands, a phenomenon known as social jetlag [8]. Several studies have associated chronotype, especially eveningness, with eating disorders and maladaptive dietary patterns, including snacking behavior [11], emotional eating [12], impaired self-regulation of eating behavior [13], and binge-eating symptoms [14,15].

As chronotype seems to be involved in regulating alimentary and behavioral patterns, we sought to investigate whether eveningness and other related sleep disruptions would be associated with binge-eating symptoms severity in individuals with obesity. Therefore, this study aimed to evaluate the relationship between chronotype and binge-eating symptoms in a sample of bariatric surgery candidates. Patients undergoing preoperative evaluation for bariatric surgery represent a subgroup of adults with severe obesity who frequently present with disordered eating patterns, sleep disturbances, and psychiatric comorbidities, all of which may influence postoperative outcomes. A more refined assessment of behavioral and chronobiological factors, such as chronotype, in this population is clinically relevant, as it may inform preoperative risk stratification, postoperative planning, and the development of individualized sleep- and behavior-related recommendations tailored to each patient’s characteristics [16].

## 2. Materials and Methods

### 2.1. Participants and Study Design

This study is a cross-sectional analysis of the relationship between binge-eating symptoms and chronotype, based on a convenience sample of bariatric surgery candidates. The sample comprised consecutive bariatric surgery candidates at a specialized obesity surgery clinic in São Paulo, Brazil (*Clínica Barimais—Medicina Integrada*). All patients fulfilled the required criteria for undergoing bariatric surgery according to Brazil’s National Supplementary Health Agency, which requires candidates to meet one of the following criteria: (i) a Body Mass Index (BMI) ≥ 40 kg/m^2^, even in the absence of comorbidities; or (ii) a BMI between 35 and 39.9 kg/m^2^ accompanied by at least one obesity-related comorbidity (e.g., type 2 diabetes, hypertension, sleep apnea, dyslipidemia, disabling joint diseases, cardiovascular disease, polycystic ovary syndrome, non-alcoholic fatty liver disease, endometriosis, rheumatic diseases, or difficult-to-control asthma). In addition, the agency requires candidates to undergo evaluation by at least four specialists: an endocrinologist, a psychologist, a nutritionist, and a cardiologist. Data were collected from the patients’ medical records, including only pre-surgical information. The inclusion criterion was patients of both sexes undergoing evaluation for bariatric surgery at the clinic. No specific inclusion criteria related to BMI or other anthropometric variables, although the site of recruitment conditionally restricts the participants to BMI values compatible with obesity. The exclusion criteria were individuals with medical record information restricted to post-surgical evaluations, or with missing information related to any of the three main outcome domains evaluated (binge-eating symptoms assessment, subjective sleep and chronotype, and mood and anxiety assessments). For individuals with pre-surgical evaluations, only the time point closest to the surgical procedure was used.

The sample size calculation was performed based on the effect size value obtained by Harb et al. [17] in their study of the correlation of binge-eating symptoms and chronotype. The calculation was performed using G-Power software (version 3.1.9.7), with values set at α = 0.05 and β = 0.20, resulting in a required sample size of 68 participants.

This study and all included procedures were approved by the Research Ethics Committee at the Universidade Federal de São Paulo (approval number #5.760.678/2022), and participants read and signed an informed consent form before the research teams received access to their medical records.

### 2.2. Procedures

Data for the study were collected between May 2019 and July 2022. It should be noted that access to this data was granted retrospectively. All data collection tools and procedures are part of the clinic’s preoperative standard care routine and were not modified for the purposes of this study. These tools included questionnaires, which were collected remotely through online forms, and all clinical data, which were gathered during appointments with the multiprofessional team. All patients seeking surgical treatment for obesity underwent evaluations and were subsequently referred to a team of specialists, including an endocrinologist, a cardiologist, a pulmonologist, a nutritionist, a psychologist, and a physiotherapist specializing in sleep. The following assessment tools were used:

Medical record data: Included information related to age, sex, body mass index (BMI), date of data collection, and date of surgery was used. BMI was categorized as follows: underweight (<18.5 kg/m^2^), eutrophic (18.5 to 24.9 kg/m^2^), overweight (25 to 29.9 kg/m^2^), obesity class I (30 to 34.9 kg/m^2^), obesity class II (35 to 39.9 kg/m^2^), and obesity class III (>40 kg/m^2^). The evaluation of candidates for bariatric surgery included a polysomnography exam (PSG) (the results of this specific exam are presented in Supplementary Material, Table S1).

Binge-Eating Scale (BES) [18,19]: This is a self-administered questionnaire used to discriminate individuals according to binge-eating symptoms severity. It consists of 16 items related to binge-eating symptoms, each scored from 0 (absence) to 3 (maximum severity). The final score is the sum of the points, and the higher the score, the greater the severity of the binge-eating behavior.

Depression, Anxiety, and Stress Scale (DASS-21) [20]: This is a questionnaire with 21 questions, which measures the intensity of behaviors and sensations experienced in the last seven days. It results in three scores, related to depression, anxiety, and stress, each being categorized into five severity levels (from normal to extremely severe).

Pittsburgh Sleep Quality Index (PSQI) [21,22]: This comprises 7 components related to sleep and is used to assess subjective sleep quality. The maximum score is 21 points, and scores greater than 5 points indicate poor sleep quality.

Insomnia Severity Index (ISI) [23]: This consists of seven questions related to insomnia symptoms (the ability to initiate and maintain sleep, early awakening, sleep satisfaction, daily functioning, distress about current sleep pattern, and the perception of others about one’s sleep problems). Each question is scored from 0 to 4, resulting in a score of up to 28 points, and is categorized into 4 severity levels (from normal to severe).

Epworth Sleepiness Scale (ESS) [24]: This questionnaire is based on 8 questions addressing the propensity of someone to nap or snooze in different daytime situations, considering the last few weeks. Each question is scored from 0 to 3, resulting in a score of up to 24 points, with scores greater than 9 indicating excessive daytime sleepiness.

Morningness and Eveningness Questionnaire (MEQ) [25,26]: This consists of 19 questions about items related to chronotype. It results in a score from 16 to 86 points, being categorized as follows: definitive evening-type (16, 30), moderate evening-type (31, 41), intermediate (42, 58), moderate morning-type (56, 69), or definitive morning-type (70, 86).

Functional Outcomes of Sleep Questionnaire (FOSQ) [27]: This questionnaire is intended to evaluate the quality of life related to sleep disorders. It evaluates five domains (activity levels, vigilance, intimacy and sexual relationships, productivity, and social outcomes), each scored from 5 to 20. Higher scores indicate better functional status.

Berlin questionnaire [28]: This consists of three domains related to the risk of having obstructive sleep apnea (OSA): snoring, daytime functioning (fatigue and history of driving accidents), and co-morbidities (obesity and hypertension). Individuals with at least two positive domains are considered at high risk for OSA.

### 2.3. Statistical Analysis

Continuous variables were tested for normality using the Shapiro–Wilk test. The association between chronotype and the continuous variables was analyzed by one-way analysis of variance (ANOVA) followed by the Games–Howell test, for variables with parametric distribution, or the Kruskal–Wallis test followed by the Dwass–Steel–Critchlow–Fligner test for variables with non-parametric distribution. The association between chronotype and categorical variables was evaluated using the X^2^ test. Finally, a multivariate linear regression was conducted, controlling for age, sex, BMI, insomnia severity, and sleep quality. Subsequently, a moderation analysis was performed to evaluate the moderating effect of chronotype on the relationship between depression, anxiety, and stress (predictor variables) and binge-eating symptoms scores (dependent variable). Continuous variables are presented as mean ± standard deviation (SD), and categorical variables are presented as frequency and percentage. All analyses were performed using the Jamovi 2.6.17 software, and the significance level was set at *p* < 0.05.

## 3. Results

### 3.1. Sample Description and Chronotype

The complete sample consisted of 100 adult bariatric surgery candidates with valid data for the three main research domains (binge-eating symptoms assessment, subjective sleep and chronotype, mood and anxiety assessment). Among the sample, 19 participants (19%) were men and 81 were women. The mean age of the sample was 35.17 ± 9.14 years. The average BMI of the sample was 40.60 ± 5.16. All patients were in the BMI range compatible with obesity, and among them, 2 participants were classified as obesity category I, 44 as obesity II, and 39 as obesity III. BMI data were unavailable for 15 participants.

**Figure 1:**
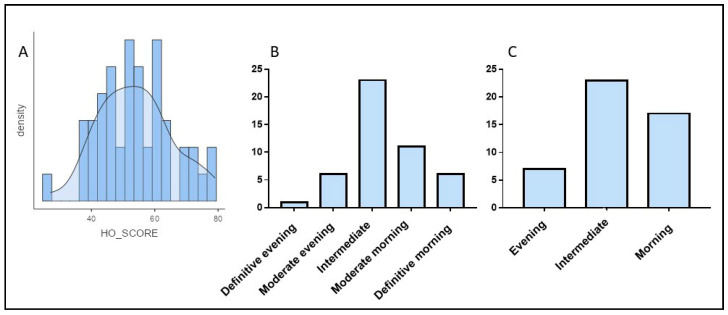
Chronotype classification based on the Morning–Eveningness Questionnaire (MEQ). (**A**): Histogram displaying the distribution of MEQ scores in the sample. (**B**): Chronotype distribution considering five categories (definitive evening-type, moderate evening-type, intermediate, moderate morning-type, definitive morning-type). (**C**): Chronotype distribution after regrouping participants into three broader categories (evening-type, intermediate, and morning-type) due to the low frequency of definitive chronotypes.

The mean MEQ score for the total sample was 53.73 ± 11.42. Based on standard MEQ cutoffs, 1 participant was considered a definitive evening-type, 15 moderate evening-type, 45 intermediate, 32 moderate morning-type, and 7 definitive morning-type. Given the low frequency of definitive chronotypes, participants were regrouped into three chronotype categories: evening-type, intermediate-type, and morning-type (Figure 1). All analyses presented below were conducted using this three-group classification.

Participants in the morning-type group (39.00 ± 9.06 years) were older than the evening-type (30.64 ± 9.59 years) and the intermediate groups (33.64 ± 7.98 years). No statistically significant differences were observed in BMI or sex distribution among chronotypes. Descriptive data for the whole sample and stratified per chronotype can be found in Table 1.

### 3.2. Subjective Sleep Assessment

The evaluation of the subjective sleep parameters indicated an overall poor sleep profile in this clinical sample. Most participants reported excessive daytime sleepiness (*n* = 51), clinically relevant insomnia symptoms (*n* = 65), with mild insomnia being the most prevalent category (*n* = 38). In addition, poor subjective sleep quality was observed in 80 participants, and 93 participants were classified as being at high risk for obstructive sleep apnea OSA.

No statistically significant associations were observed between chronotype groups (evening-type, intermediate-type, and morning-type) and any of the subjective sleep assessment measures, including daytime sleepiness, insomnia severity, sleep quality, functional outcomes of sleep, or OSA risk (Table 2 and Table 3).

### 3.3. Assessment of Depression, Anxiety, and Stress

Most participants did not present clinically relevant symptoms of stress (*n* = 84), anxiety (*n* = 84), or depression (*n* = 87), according to DASS-21 severity classifications. No statistically significant differences were observed across chronotype groups for depression, anxiety, or stress scores, indicating that psychological symptom burden was comparable among evening-, intermediate-, and morning-type individuals (Table 4).

### 3.4. Chronotype and Binge-Eating Symptoms

**Figure 2:**
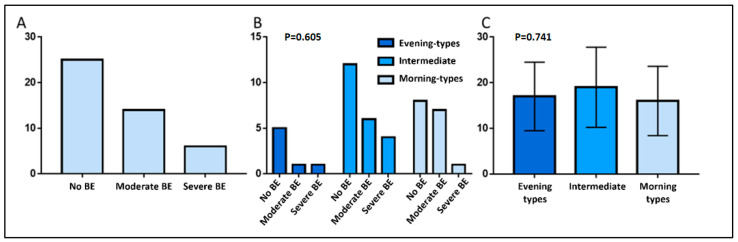
Binge-eating symptoms and chronotype. (**A**): Distribution of participants classified as having no, moderate, or severe binge-eating symptoms. (**B**): Distribution of binge-eating symptoms severity across morning-, intermediate-, and evening-type chronotypes. (**C**): Binge-eating symptoms scores by chronotype (mean ± standard deviation). BE—binge eating.

Half of the sample (*n* = 50) had a score compatible with binge-eating behavior, with 38 participants classified as having moderate symptoms and 12 as having severe symptoms (Figure 2A). The proportion of individuals with moderate or severe binge-eating symptoms was similar across the three chronotypes (*p* = 0.794), with individuals with no binge-eating symptoms accounting for around 50% of each group (Figure 2B). No significant association was observed between chronotype and binge-eating score (*p* = 0.702—Figure 2C).

Across all models, stress, anxiety, and depression were not significant predictors of binge-eating scores after adjusting for covariates (sex, age, BMI, insomnia symptoms, and sleep quality) (Table 5). Similarly, chronotype did not show significant main effects on binge eating in any comparison (morning–evening, morning–intermediate, or intermediate–evening types). Interaction terms between chronotype and each psychological factor (stress, anxiety, and depression) were also non-significant, indicating no moderating effect of chronotype on the association between psychological symptoms and binge-eating severity.

Overall, the 95% confidence intervals for all predictors and interaction terms included zero, and *p*-values remained above the conventional significance threshold, indicating that neither psychological symptoms nor chronotype—independently or in interaction—were associated with binge-eating symptom severity in this clinical sample.

## 4. Discussion

In the present study involving bariatric surgery candidates, we found a prevalence of 50% of binge-eating symptoms. This is in line with previous studies that demonstrate a high prevalence of binge-eating behavior among individuals with obesity [3,4,29], although being slightly lower than the level reported in a previous Brazilian study (68%) among individuals living with obesity in a population-based sample [1]. This difference in prevalence estimates might be due to the use of different diagnostic criteria, as the previous study used DSM-5 to evaluate BED, while the present study used the BES [18,19]. Despite this, both studies indicate that the prevalence of binge-eating behavior is high in individuals with obesity.

In an attempt to better understand the possible behavioral components related to binge-eating behavior, we evaluated chronotype in our sample, as some studies have reported that having an evening-type chronotype is more strongly associated with psychiatric co-morbidities, including eating disorders, than with other chronotypes [14,17,30,31]. In our sample, 16% of patients were evening-type, 45% intermediate type, and 39% morning-type, and the chronotypes were not associated with binge-eating symptoms. It should be noted that our sample was entirely composed of individuals with obesity, all of whom were candidates for bariatric surgery, with an average BMI of 40. The use of samples of bariatric surgery candidates for studies about dietary patterns is common, as it restricts the sample to individuals at high risk of eating disorders [3,4,32].

Previous studies have related chronotype to binge-eating behavior, usually associating it with eveningness [14,17,33,34]. Among the main explanations for the associations of evening-type individuals with binge-eating behavior are the following: (1) a delayed feeding window, with a greater evening food intake; (2) behavioral changes caused by sleep deprivation (often seen in evening-type individuals due to social jetlag), including disinhibition, impulsivity, and anxiety; and (3) changes in hormonal levels associated with appetite control due to sleep deprivation, including ghrelin and leptin.

The current study produced contrary results, finding no association between chronotype and binge-eating behavior. We suggest three possible explanations for this: (1) the proportion of individuals with obesity in the sample, (2) the sex ratio in the sample, and (3) the role of psychological features in the development of binge-eating behavior.

In respect of the first explanation, a study by Harb et al. [17] found a correlation between binge-eating behavior and chronotype, reporting that eveningness was correlated with higher BES scores in subjects older than 40 years. However, their sample included mainly individuals without obesity (18% obese, 48% overweight, and 34% eutrophic participants). However, our sample was 100% composed of participants living with obesity (of which 98% were classified as obesity II or III). Bariatric surgery candidates typically report long trajectories of repeated dieting and weight regain; patterns consistently linked to increased risk of binge-eating behaviors [35]. Recurrent dieting can influence appetite regulation, cognitive restraint, and loss-of-control eating, which may exert a strong effect on binge-eating severity. Because these dieting histories tend to be highly prevalent and influential among individuals seeking bariatric surgery, they may have contributed to homogenizing binge-eating patterns across chronotypes. Since dieting history was not collected, future studies should evaluate this factor as a potential determinant of binge eating in this population.

Another possible explanation for the lack of association between chronotype and binge-eating symptoms is the sex ratio in our sample. A previous study by Amicis et al. [36] found that the association between chronotype and binge-eating symptoms is only significant among men and reported a remarkable negative correlation between BES and MEQ scores. However, no such correlation was observed among women. As our sample was predominantly composed of women (81%), the potential for observing these effects was limited.

Finally, our sample was composed of individuals with low levels of depression, anxiety, and stress. Considering that these psychological traits might mediate the relationship between chronotype and binge-eating behavior, their low prevalence might have affected the results.

One possible limitation of the current study, and a further potential explanation of our results, was the low prevalence of definitive (or extreme) chronotypes, especially the definitive evening-type. If extreme chronotypes were better represented in our sample, the likelihood of finding significant associations might have increased. The analysis, which allocated the participants into three chronotype groups, was an attempt to control this, but no significant results were found.

It is also important to acknowledge that the use of the BES represents a methodological limitation when interpreting the prevalence of binge-eating symptoms in our sample. The BES is a screening instrument that quantifies the severity of binge-eating symptoms, but it does not provide a formal diagnosis of BED according to DSM-5 criteria, which require the presence of objective binge episodes, loss of control, marked distress, and a minimum frequency and duration. Consequently, the prevalence observed in our study reflects symptom severity rather than diagnostic-level BED, which may partially explain discrepancies with studies reporting BED prevalence using structured clinical interviews. This distinction is relevant for interpreting our null findings, as symptom-based scales may capture a broader and more heterogeneous phenotype, possibly diluting the associations between chronotype and clinically defined BED. Another possible limitation was the way the patients reported their symptoms in the pre-surgical evaluation. Bariatric surgery candidates undergo an intense pre-surgical evaluation, and some results may lead to the postponement of the surgery. As previously described by other authors, bariatric candidates may hide or “soften” information to avoid having their surgery delayed or not performed [37]. Finally, another important limitation of this study is the inability to compare data on binge eating and chronotype with a population without obesity, since the impulsive component may be related to chronotype rather than to obesity itself.

Our results corroborate the high prevalence of binge-eating behavior among individuals with obesity, although no significant associations between chronotype and binge-eating severity symptoms were found. We recommend that future studies investigate the longitudinal relationship between chronotype and binge-eating behavior among bariatric surgery candidates, especially focusing on post-surgical outcomes. Important aspects to be evaluated include possible changes in eating behavior patterns after surgery, which may be different among individuals with different chronotypes, and the possible effect of the chronotype on the maintenance or regain of weight.

In conclusion, binge-eating behavior is highly prevalent among obese individuals, especially among candidates for bariatric surgery. However, there is no association between chronotype and binge-eating symptoms in our sample. Further studies are needed to explore the roles of obesity, sex, and psychological characteristics to assess the possible associations between these chronotypes and binge-eating behavior.

## Figures and Tables

**Table 1:** Descriptive data of the total sample by chronotype group.

	Total Sample			Evening-Type			Intermediate			Morning-Type			
	*n* (%)	Mean	SD	*n* (%)	Mean	SD	*n* (%)	Mean	SD	*n* (%)	Mean	SD	*p*
Age	85 (100)	35.17	9.14	14 (16.5)	30.64	9.59	39 (45.9)	33.64	7.99	32 (37.6)	39.00	9.06	0.012
Weight (kg) *	84 (100)	113.14	19.56	14 (16.7)	115.03	22.87	39 (46.4)	115.90	18.64	31 (36.9)	108.82	19.01	0.184
BMI (kg/m^2^) *	84 (100)	40.60	5.16	14 (16.7)	41.21	5.36	39 (46.4)	40.90	5.05	31 (36.9)	39.95	5.31	0.556
Obesity I	2 (100)			0			1 (50)			1 (50)			0.955
Obesity II	43 (100)			8 (18.6)			19 (44.2)			16 (37.2)			
Obesity III	39 (100)			6 (15.4)			19 (48.7)			14 (35.9)			
Sex													
Female	81 (100)			12 (14.8)			35 (43.2)			34 (41.9)			0.439
Male	19 (100)			4 (21.0)			10 (52.6)			5 (26.3)			

*—Data with non-parametric distribution, according to post hoc test (*p* < 0.05). Sample size is smaller than 100 for most variables due to missing data. BMI—Body mass index; SD—standard deviation.

**Table 2:** Subjective sleep assessment in total sample and according to chronotype group—numerical outcomes.

	*n* (%)	Mean	SD	*n* (%)	Mean	SD	*n* (%)	Mean	SD	*n* (%)	Mean	SD	*p*
ESS—Sleepiness *	100 (100)	10.09	6.09	16 (16)	11.31	5.52	45 (45)	10.31	6.30	39 (39)	9.33	6.10	0.506
ISI—Insomnia symptoms *	100 (100)	10.07	6.22	16 (16)	10.25	5.79	45 (45)	10.20	6.31	39 (39)	9.85	6.43	0.868
PSQI—Sleep quality	100 (100)	9.33	4.16	16 (16)	9.69	3.88	45 (45)	9.56	4.19	39 (39)	8.92	4.30	0.741
FOSQ—Productivity *	100 (100)	3.21	0.66	16 (16)	3.07	0.63	45 (45)	3.21	0.68	39 (39)	3.25	0.64	0.551
FOSQ—Social *	100 (100)	3.12	0.95	16 (16)	3.00	0.95	45 (45)	3.02	0.96	39 (39)	3.27	0.92	0.338
FOSQ—Activity *	100 (100)	2.87	0.68	16 (16)	2.67	0.72	45 (45)	2.82	0.61	39 (39)	3.01	0.71	0.113
FOSQ—Vigilance *	100 (100)	3.11	0.75	16 (16)	3.05	0.79	45 (45)	3.08	0.75	39 (39)	3.16	0.75	0.856
FOSQ—Sexual *	100 (100)	3.13	0.82	16 (16)	2.84	0.99	45 (45)	3.13	0.84	39 (39)	3.24	0.72	0.381
FOSQ—General *	100 (100)	15.43	3.07	16 (16)	14.63	3.23	45 (45)	15.27	3.02	39 (39)	15.93	3.05	0.282

*—Data with non-parametric distribution. SD—standard deviation; ESS—Epworth Sleepiness Scale; FOSQ—Functional Sleep Outcomes Questionnaire; ISI—Insomnia Severity Index; PSQI—Pittsburgh Sleep Quality Index.

**Table 3:** Subjective sleep assessment in total sample and chronotype groups—categorical outcomes.

	Total Sample	Evening-Types		Intermediate		Morning-Types		
	*n* (%)	*n*	%	*n*	%	*n*	%	*p*
ESS—Sleepiness								
Normal	49 (49)	5	10.20%	22	44.90%	22	44.90%	0.238
Excessive daytime sleepiness	51 (51)	11	21.57%	23	45.10%	17	33.33%	
ISI—Insomnia symptoms								
Normal	35 (35)	5	14.29%	17	48.57%	13	37.14%	0.868
Mild	38 (38)	6	15.79%	15	39.47%	17	44.74%	
Moderate	23 (23)	5	21.74%	11	47.83%	7	30.43%	
Severe	4 (4)	0	0.00%	2	50.00%	2	50.00%	
PSQI—Sleep quality								
Normal	18 (18)	2	11.11%	8	44.44%	8	44.44%	0.478
Poor sleep quality	80 (80)	14	17.50%	37	46.25%	29	36.25%	
Berlin questionnaire								
Low risk for OSA	7 (7)	1	14.29%	3	42.86%	3	42.86%	0.975
High risk for OSA	93 (93)	15	16.13%	42	45.16%	36	38.71%	

ESS—Epworth Sleepiness Scale; FOSQ—Functional Sleep Outcomes Questionnaire; ISI—Insomnia Severity Index; OSA—Obstructive sleep apnea; PSQI—Pittsburgh Sleep Quality Index. Sample size is smaller than 100 for PSQI due to missing data.

**Table 4:** Stress, anxiety, and depression symptoms among chronotypes.

	Total Sample			Evening-Types			Intermediate			Morning-Types			
	*n* (%)	Mean	SD	*n* (%)	Mean	SD	*n* (%)	Mean	SD	*n* (%)	Mean	SD	*p*
DASS—Stress *	100 (100)	6.63	4.17	16 (100)	7.37	5.17	45 (100)	6.18	3.47	39 (100)	6.85	3.47	0.755
Normal	84 (84)			12 (75)			40 (88.9)			32 (82.0)			0.511
Mild	14 (14)			3 (18.7)			5 (11.1)			6 (15.4)			
Moderate	2 (2)			1 (6.3)			0 (0)			1 (2.6)			
Severe	0 (0)			0 (0)			0 (0)			0 (0)			
DASS—Anxiety *	100 (100)	3.21	3.43	16 (100)	3.69	3.89	45 (100)	2.84	3.89	39 (100)	3.44	3.58	0.778
Normal	84 (84)			14 (87.5)			41 (91.1)			29 (74.4)			0.113
Mild	9 (9)			0 (0)			2 (4.4)			7 (17.9)			
Moderate	6 (6)			2 (12.5)			1 (2.2)			3 (7.7)			
Severe	1 (1)			0 (0)			1 (2.2)			0 (0)			
DASS—Depression *	100 (100)	4.64	4.21	16 (100)	7.00	5.55	45 (100)	4.27	3.54	39 (100)	4.10	4.10	0.095
Normal	87 (87)			13 (81.3)			41 (91.1)			33 (84.6)			0.635
Mild	7 (7)			1 (6.3)			2 (4.4)			4 (10.3)			
Moderate	6 (6)			2 (12.5)			2 (4.4)			2 (5.1)			
Severe	0 (0)			0 (0)			0 (0)			0 (0)			

*—Data with non-parametric distribution. DASS—Depression, Anxiety, and Stress Scale; SD—standard deviation.

**Table 5:** Linear regression models including chronotype interactions for predicting binge-eating severity symptoms.

	β	β 95% Confidence Intervals		*p*
		Lower	Upper	
Stress as predictor of binge-eating symptoms				
Intercept	0.346	−0.295	0.988	<0.001
Covariates				
Sex (Male–Female)	−0.560	−1.172	0.051	0.072
Age	−0.013	−0.253	0.227	0.915
BMI	−0.054	−0.285	0.177	0.644
ISI—Insomnia symptoms	0.128	−0.187	0.443	0.421
PSQI—Sleep quality	0.025	−0.286	0.335	0.874
Predictor variables				
Chronotype (Intermediate–Evening-type)	−0.187	−0.874	0.500	0.589
Chronotype (Morning-type–Intermediate)	−0.235	−0.736	0.267	0.354
Chronotype (Morning-type–Evening-type)	−0.367	−1.101	0.366	0.321
DASS—Stress	0.002	−0.428	0.433	0.992
Moderation				
Chronotype (Intermediate–Evening-type) × DASS—Stress	0.074	−0.495	0.643	0.796
Chronotype (Morning-type–Intermediate) × DASS—Stress	−0.162	−0.683	0.360	0.538
Chronotype (Morning-type–Evening-type) × DASS—Stress	0.291	−0.284	0.865	0.317
Anxiety as predictor of binge-eating symptoms				
Intercept	0.345	−0.232	0.921	<0.001
Covariates	−0.557	−1.172	0.057	0.075
Sex (Male–Female)	−0.036	−0.281	0.209	0.770
Age	−0.030	−0.270	0.210	0.802
BMI	0.151	−0.185	0.487	0.373
ISI—Insomnia symptoms	0.029	−0.289	0.348	0.854
PSQI—Sleep quality	−0.557	−1.172	0.057	0.075
Predictor variables				
Chronotype (Intermediate–Evening-type)	−0.203	−0.833	0.426	0.522
Chronotype (Morning-type–Intermediate)	−0.215	−0.721	0.291	0.399
Chronotype (Morning-type–Evening-type)	−0.418	−1.106	0.270	0.230
DASS—Anxiety	−0.090	−0.591	0.411	0.721
Moderation				
Chronotype (Intermediate–Evening-type) × DASS—Anxiety	0.054	−0.544	0.652	0.858
Chronotype (Morning-type–Intermediate) × DASS—Anxiety	0.225	−0.267	0.717	0.365
Chronotype (Morning-type–Evening-type) × DASS—Anxiety	0.279	−0.326	0.883	0.361
Depression as predictor of binge-eating symptoms				
Intercept	0.317	−0.267	0.901	<0.001
Covariates				
Sex (Male–Female)	−0.529	−1.143	0.086	0.091
Age	−0.021	−0.260	0.218	0.861
BMI	−0.052	−0.295	0.192	0.673
ISI—Insomnia symptoms	0.143	−0.172	0.458	0.370
PSQI—Sleep quality	−0.001	−0.309	0.307	0.997
Predictor variables				
Chronotype (Intermediate–Evening-type)	−0.157	−0.790	0.476	0.623
Chronotype (Morning-type–Intermediate)	−0.180	−0.682	0.322	0.476
Chronotype (Morning-type–Evening-type)	−0.391	−1.084	0.301	0.264
DASS—Depression	0.075	−0.364	0.515	0.733
Moderation				
Chronotype (Intermediate–Evening-type) × DASS—Depression	0.158	−0.404	0.721	0.577
Chronotype (Morning-type–Intermediate) × DASS—Depression	0.217	−0.330	0.763	0.432
Chronotype (Morning-type–Evening-type) × DASS—Depression	−0.004	−0.579	0.572	0.990

## Data Availability

The original contributions presented in this study are included in the article/Supplementary Materials. Further inquiries can be directed to the corresponding author.

## Supplementary Materials

The following supporting information can be downloaded at: https://www.mdpi.com/article/10.3390/jpm16010037/s1, Table S1: Polysomnographic results in total sample and chronotype groups.

## Abbreviations

The following abbreviations are used in this manuscript:

BMI	Body mass index
PSG	Polysomnography
BES	Binge-eating scale
DASS-21	Depression, Anxiety, and Stress Scale
PSQI	Pittsburgh Sleep Quality Index
ISS	Insomnia Severity Index
ESS	Epworth Somnolence Scale
MEQ	Morningness and Eveningness Questionnaire
FOSQ	Functional Outcomes of Sleep Questionnaire
OSA	Obstructive sleep apnea
SD	Standard deviation
