# Supplementary material for: Associations Between Binge-Eating Symptoms and Chronotype Among Bariatric Surgery Candidates: Clinical Implications for Preoperative Assessment—A Cross-Sectional Study
Source: J Pers Med. 2026 Jan 7;16(1):37. doi: 10.3390/jpm16010037 (PMC12842636; doi:10.3390/jpm16010037)
Supplement: Supplementary file 1 [file jpm-16-00037-s001.zip › jpm-3993773-supplementary.pdf]

Supplementary material

According to the objective PSG sleep analysis, the average AHI was 19.95±22.05. The prevalence of OSA in the sample was 74%. Among those affected, 30 had mild OSA, 24 moderate OSA and 20 severe OSA. There was no statistically significant association between chronotype and any PSG parameter (Table S1).

Table S1. Polysomnographic results in total sample and chronotype groups.

	**Total sample**			**Evening-types**			**Intermediate**			**Morning-types**			
	n	Mean	SD	*n*	Mean	SD	*n*	Mean	SD	*n*	Mean	SD	*p*
Sleep latency (min)*	100	34.85	38.24	16	53.15	52.82	45	36.22	41.62	39	25.75	21.80	0.553
REM latency (min)*	100	131.97	99.59	16	112.00	45.10	45	133.75	122.45	39	138.12	86.35	0.714
Total sleep time (min)*	100	387.61	303.97	16	339.06	66.18	45	360.99	67.89	39	438.25	478.74	0.275
Sleep efficiency (%)*	100	82.90	11.85	16	80.14	13.75	45	81.67	12.77	39	85.45	9.53	0.334
N1 (%)*	98	8.23	8.37	16	8.13	6.00	45	8.59	8.99	37	7.83	8.63	0.791
N2 (%)	98	55.45	11.05	16	51.11	10.03	45	56.39	11.14	37	56.20	11.19	0.194
N3 (%)	98	19.22	9.26	16	23.38	9.51	45	18.67	9.84	37	18.09	8.12	0.160
REM (%)	99	16.90	7.63	16	17.39	6.65	45	16.35	7.49	38	17.33	8.29	0.812
WASO (min)*	91	42.53	43.12	16	34.81	35.36	43	48.76	50.31	32	38.03	35.48	0.455
Arousals (n)*	99	102.52	96.69	16	97.06	36.05	44	108.68	115.48	39	97.80	91.79	0.354
Arousal index(n/h)*	99	19.50	29.04	16	16.46	4.97	44	23.69	41.42	39	16.03	13.64	0.229
Leg movements (n)*	93	6.57	39.30	16	3.74	11.85	42	11.08	56.96	35	2.46	12.61	0.071
RDI*	99	20.62	22.68	16	21.22	25.38	45	21.39	24.64	38	19.46	19.42	0.984
AHI*	100	19.95	22.05	16	21.23	25.37	45	20.11	23.54	39	19.24	19.21	0.980
SpO2 average*	96	93.19	2.13	16	93.44	1.59	44	93.48	2.35	36	92.73	2.02	0.104
SpO2 nadir*	96	82.04	8.76	16	83.50	7.54	44	82.18	9.72	36	81.22	8.14	0.354
SpO2<90%*	95	8.28	16.30	16	6.74	13.52	45	6.79	15.17	34	10.97	18.86	0.390

*: Data with non-parametric distribution. AHI: Apnea-hypopnea index. RDI: Respiratory disturbance index. REM: Rapid eye movements. WASO: Wake after sleep onset.
